# Primary adrenal CD20-negative diffuse large B-cell lymphoma: Diagnostic and therapeutic challenges—A case report

**DOI:** 10.1016/j.radcr.2025.05.072

**Published:** 2025-06-21

**Authors:** Mohammad Saad Salim Naviwala, Daania Shoaib, Reyan Hussain Shaikh, Romana Idrees, Muhammad Afzal

**Affiliations:** a Department of Medical Oncology, Aga Khan University Hospital, Karachi, Pakistan; b Medical College, Aga Khan University, Karachi, Pakistan; c Department of Pathology, Aga Khan University Hospital, Karachi, Pakistan

**Keywords:** Primary adrenal lymphoma, CD20-negative DLBCL, CHOP chemotherapy, Adrenal insufficiency, Extra-nodal lymphoma

## Abstract

Primary adrenal lymphoma (PAL) is a rare, aggressive malignancy, often manifesting as bilateral adrenal masses with insufficiency. CD20-negative diffuse large B-cell lymphoma (DLBCL), a rarer variant, exhibits resistance to standard therapies and a poorer prognosis. We report a 60-year-old woman with bilateral adrenal masses diagnosed as CD20-negative unclassifiable DLBCL. Despite an initial response to CHOP chemotherapy, her disease progressed, necessitating salvage therapy with gemcitabine, cisplatin and dexamethasone. This case highlights the diagnostic complexity of CD20-negative DLBCL, particularly when presenting in rare extra-nodal sites such as the adrenal glands. Comprehensive immunohistochemical profiling and multidisciplinary management are crucial for accurate diagnosis and treatment planning. Given the poor prognosis and lack of standardized therapies, further research is needed to refine treatment strategies and improve outcomes for patients with CD20-negative DLBCL.

## Introduction

Primary Adrenal Lymphoma (PAL) is an extremely rare entity, representing around 1% of all non-Hodgkin lymphomas and less than 3% of primary extra-nodal lymphomas [1,2], with a total of less than 250 reported cases in the literature [3]. These tumors predominantly affect individuals aged 60-70 years, exhibiting a modest male predominance [1,2,4]. Most patients have bilateral adrenal gland involvement often manifesting as adrenal insufficiency and up to 80% are diffuse large B-cell lymphomas (DLBCL) histologically [3]. Owing to the anatomic location, lymphoid tissue is absent in the adrenal glands, making the pathogenesis of PAL elusive. Leading theories about pathogenesis involve immune dysfunction, viral etiologies such as human immunodeficiency virus (HIV) and Epstein-Barr Virus (EBV), and certain genetic mutations [1,4]. PAL is particularly aggressive and prognosis remains grim, with 1 year survival placed between 17-20% [1,4].

Another unique subtype in the realm of lymphomas is the CD20-negative DLBCL, which also demonstrates a decidedly aggressive course. De novo CD20-negative DLBCL accounts for less than 1–3% of cases[5]. There are further known subtypes of CD20-negative DLBCL [6]. CD20 is involved in the processes of B cell maturation, differentiation, and activation, and it is postulated that certain genetic mutations lead to the development of the negative phenotype [5]. These tumors are associated with extra-nodal disease, unusual morphology, and a difficult clinical course. All these factors are compounded by the negative phenotype making CD20-negative DLBCL resistant to the otherwise beneficial standard chemotherapeutic regimens [7], posing a therapeutic challenge to the clinician.

Herein, we present a distinctive case of a PAL found to be de novo unclassifiable CD20-negative on histology, which after an initial period of improvement progressed with a significant disease burden.

## Case report

A 60-year-old woman, with no known co-morbidities and a European Cooperative Oncology Group (ECOG) Performance Status score of II, presented to the outpatient department with complaints of lethargy, generalized weakness with persistent nausea, and a resultant decrease in appetite. These symptoms were first noticed approximately 6 months before presentation and progressively kept increasing despite symptomatic care. No fever or other systemic symptoms were reported. This was accompanied by a subjective weight loss in the same period.

Her past medical history revealed a Total Abdominal Hysterectomy with Bilateral Salpingo-oophorectomy (TAH/BSO) 11 years ago for dysfunctional uterine bleeding. The surgical pathology for the above was negative. A probe into her family history revealed that her mother had suffered from and succumbed to endometrial carcinoma. There was no history of any substance abuse. On examination, the patient measured approximately 162 cm in height and weighed 62 kg, corresponding to a BMI of 23.6 kg/m². Her vital signs were within normal limits, and systemic examination revealed no significant findings.

**Fig. 1:**
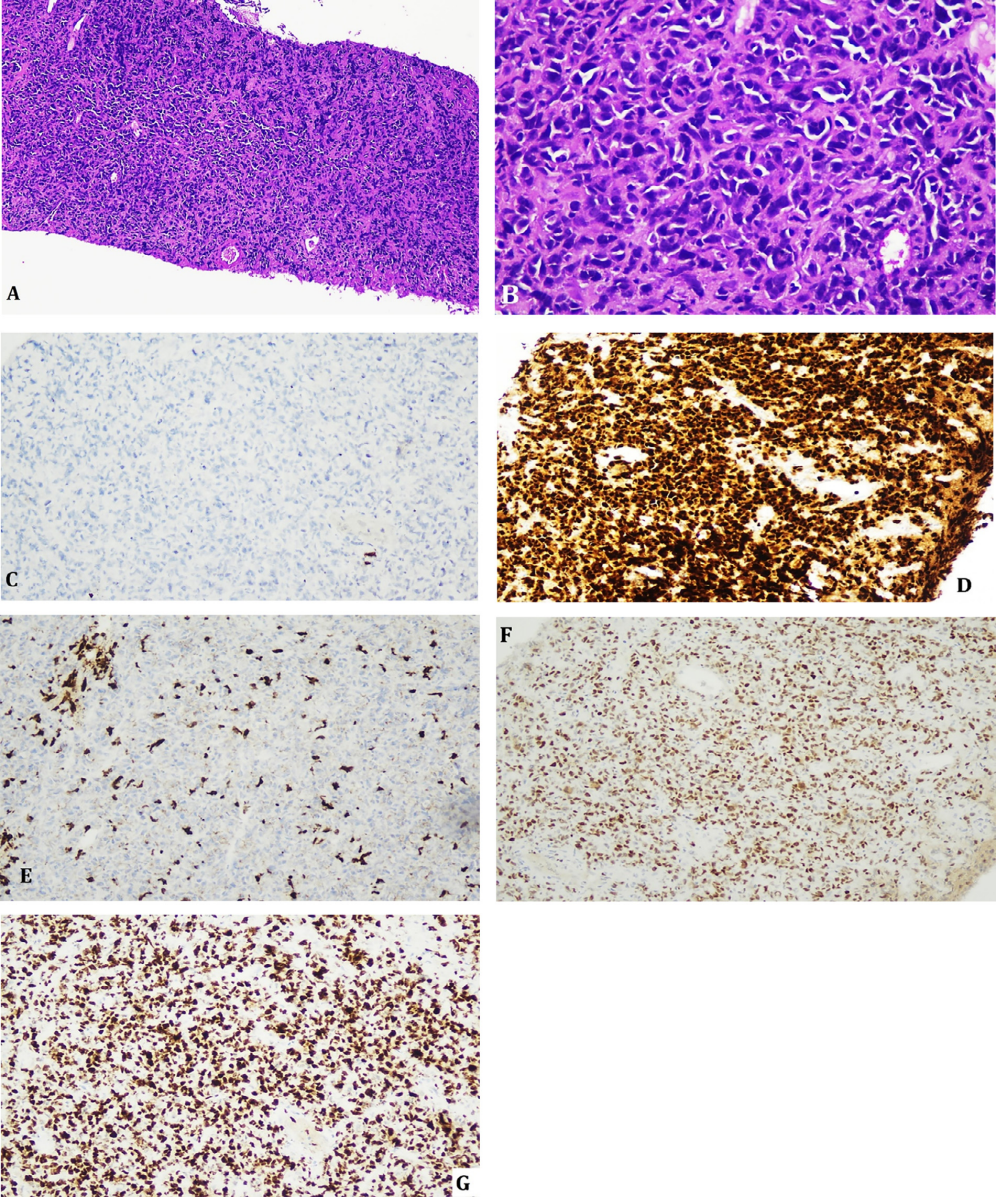
Histopathological images. (A) Low-power view (4x) displaying section of the adrenal mass showing sheets of diffuse infiltration of large lymphoid cells (H&E). (B) High-power view (40x) showing large cells with hyperchromatic nuclei. (C) CD20 – negative staining. (D) PAX-5 positivity. (E) Occasional CD3 staining. (F) CD30 – negative staining. (G) Markedly raised Ki67.

She had brought along a contrast-enhanced Computed Tomography (CT) scan of the abdomen conducted at another healthcare facility, which revealed bilateral adrenal masses. The left adrenal lesion measured 6.8 × 5.0 cm and the right measured 6.5 × 3.0 cm. Both appeared hypodense, with well-defined yet mildly irregular margins, and showed mild heterogeneous postcontrast enhancement. No calcifications or hemorrhagic components were noted. Adjacent structures including the liver, kidneys, and inferior vena cava appeared uninvolved. A few enlarged para-aortic lymph nodes were seen, the largest measuring 1.1 cm, suggestive of regional nodal involvement. No evidence of necrosis or vascular invasion was detected on initial imaging. The original CT images, however, were unavailable for inclusion. Clinically, she had persistent hypotension with blood pressures ranging around 90/60 mmHg. Laboratory findings showed mild hyponatremia (serum sodium 132 mmol/L) consistent with adrenal insufficiency. A referral to an Endocrinologist was made and investigations revealed low cortisol levels of 70.90 nmol/L (132.97-537.93 nmol/L) and raised adrenocorticotropic hormone (ACTH) levels of 12.2 pmol/L (0-10.13 pmol/L). Plasma-free metanephrines were found to be within normal range. Thus, corticosteroid replacement was duly started for symptom control and she simultaneously underwent an image-guided biopsy of the left adrenal gland. Histopathology revealed sheets of medium to large atypical lymphoid cells. Immunohistochemical stains revealed CD20 to be negative. PAX-5 and CD79a were found to be positive (Fig. 1A-G). A diagnosis of high-grade B-cell non-Hodgkin Lymphoma was made. Further immunohistochemical testing revealed BCL-2, C-myc, and BCL-6 positivity, but unfortunately, further testing for rearrangement by fluorescence in-situ hybridization (FISH) was not possible due to exhaustion of tissue.

**Fig. 2 (A and B):**
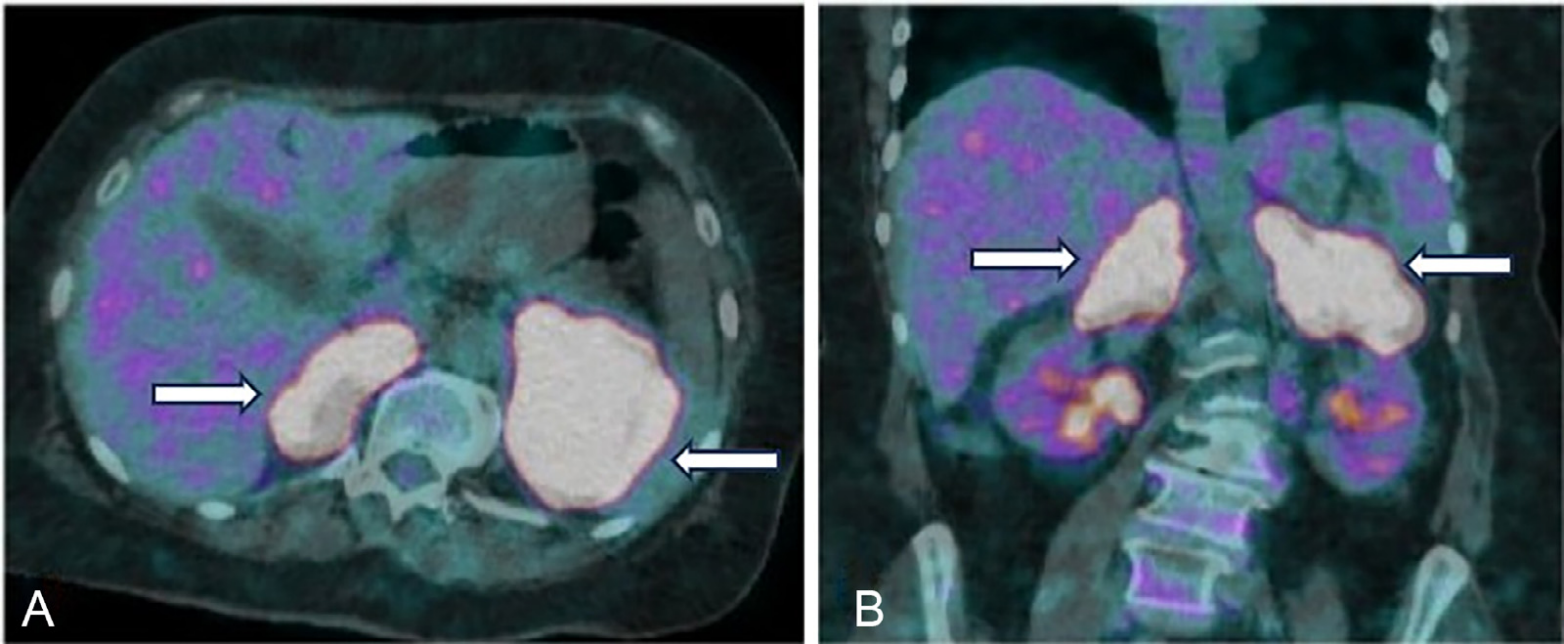
Axial and coronal fusion images from baseline PET-CT at diagnosis, showing hypermetabolic bilateral adrenal masses denoted by arrows, consistent with biopsy-proven lymphoma.

On PET/CT, both adrenal lesions demonstrated markedly increased FDG uptake—left: SUVmax 32.8 and right: SUVmax 29.8—exhibiting a homogeneous pattern of hypermetabolism without central necrosis. These imaging features strongly favored a diagnosis of lymphoma. Additionally, mild FDG uptake was observed in bilateral para-aortic lymph nodes (SUVmax ∼2.3), with no significant mediastinal or pulmonary involvement. Collectively, the findings were consistent with Stage IE primary adrenal lymphoma (Fig. 2A and B).

Relevant investigations showed mildly elevated uric acid and lactate dehydrogenase (LDH) levels (0.40 mmol/L and 5.65 µkat/L, respectively). Viral markers including HIV assay were negative.

Given the patient’s poor ECOG performance status, a dose-adjusted EPOCH regimen was initially considered due to its efficacy in aggressive lymphomas. However, after a thorough discussion about the potential toxicities and based on the patient's and family's concerns, this option was declined. Consequently, a less intensive yet effective approach was adopted using CHOP (Cyclophosphamide, Doxorubicin, Vincristine, and Prednisolone) in combination with high-dose methotrexate (3 g/m²) to address the risk of central nervous system (CNS) involvement. Intrathecal methotrexate was administered with each cycle as CNS prophylaxis. Cerebrospinal fluid cytology was negative for malignant cells.

**Fig. 3 (A and B):**
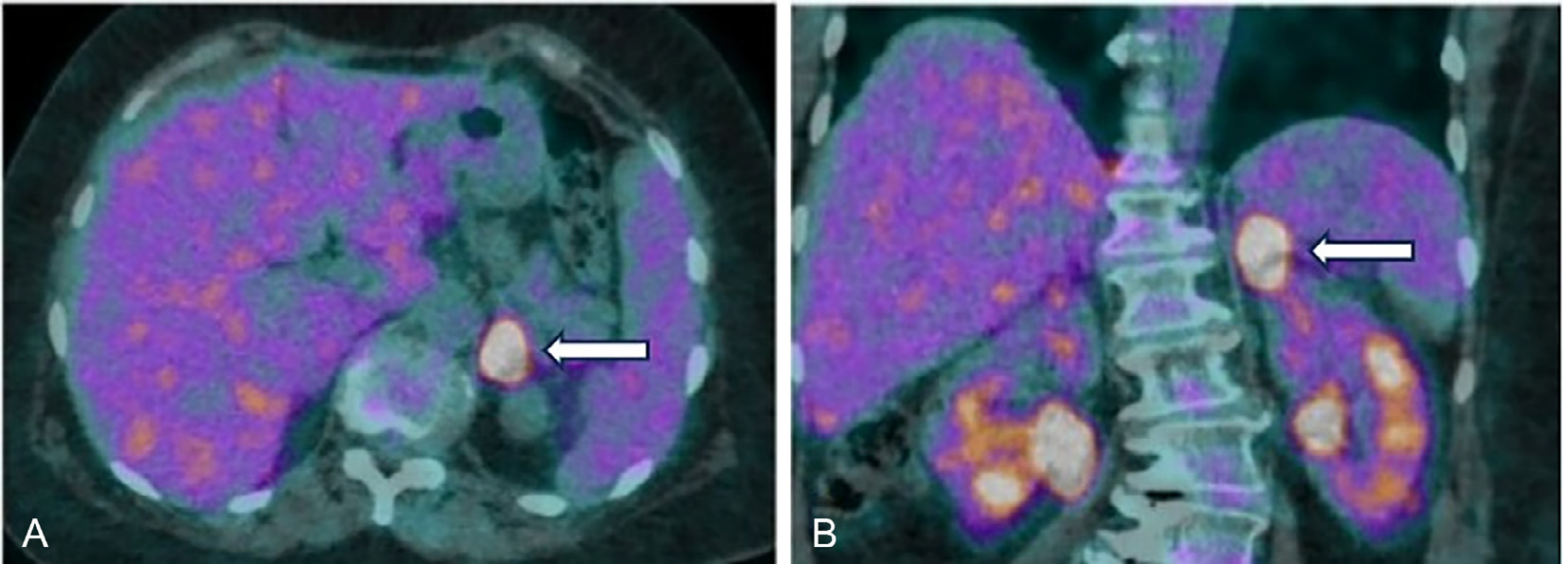
Axial and coronal fusion images from interim PET-CT after 4 cycles of CHOP and intrathecal methotrexate. Imaging reveals interval resolution of the hypermetabolic right adrenal mass and significant reduction in size and metabolic activity of the left adrenal mass, with a small residual lesion over the medial and lateral limbs.

She underwent an interim PET scan after receiving 4 cycles of CHOP with intrathecal methotrexate, which revealed a partial metabolic response—characterized by normalization of the right adrenal mass and reduction of the left adrenal mass to 20 × 18 mm (SUVmax: 14.8). The interim scan corresponded to a Deauville score of 4, indicating residual but reduced metabolic activity (Fig. 3A and B). Due to the response observed, she received 2 more cycles of CHOP, and this was followed by 2 cycles of high-dose methotrexate (3 g/m²) once cell counts had recovered.

**Fig. 4 (A and B):**
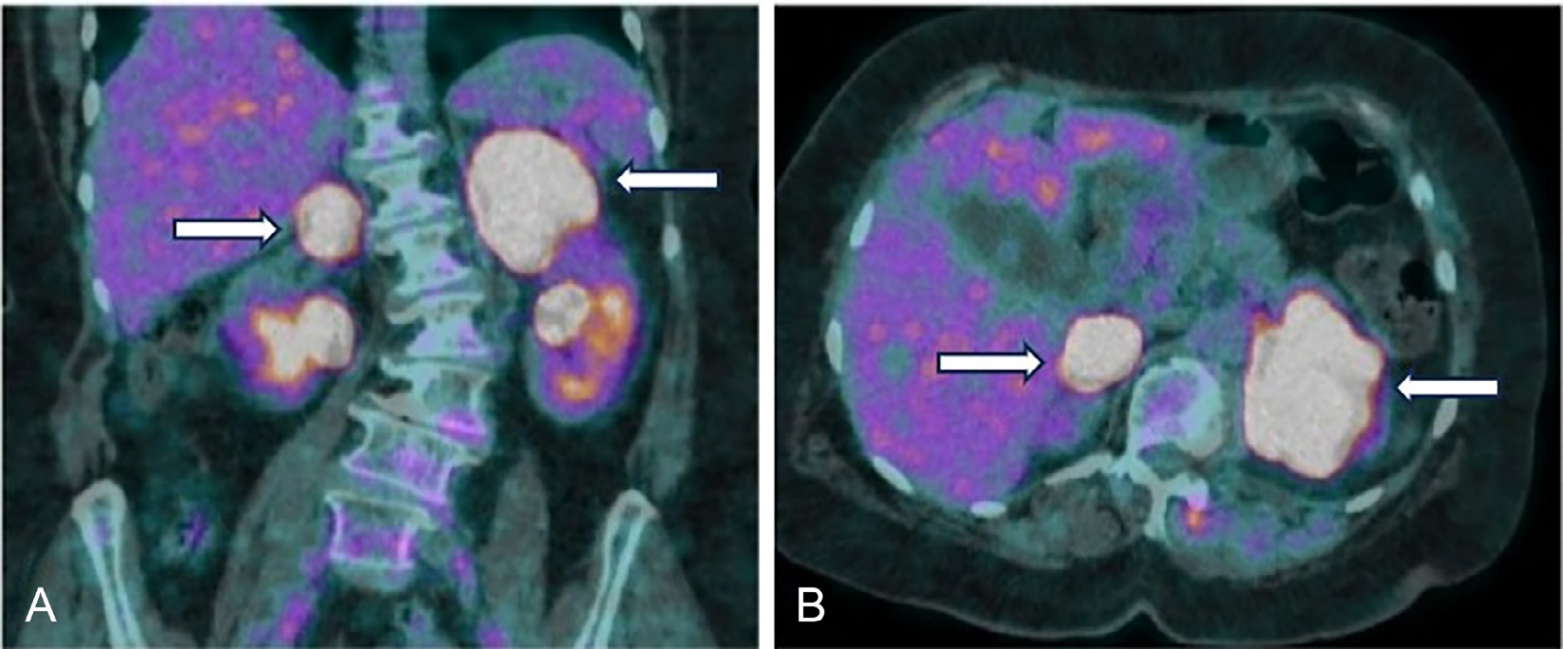
Axial and coronal fusion images from end-of-treatment PET-CT after 6 cycles of CHOP and 2 cycles of high-dose methotrexate. Imaging demonstrates interval progression in size and metabolic activity of bilateral adrenal masses, representing metabolically progressive disease and treatment failure.

A PET-CT scan performed 1 month after completing the treatment regimen revealed disease progression. The scan demonstrated a significant increase in both the size and metabolic activity of the bilateral adrenal masses, with the right adrenal lesion measuring 38 × 35 mm (SUVmax 43.8) and the left adrenal lesion measuring 66 × 56 mm (SUVmax 35.4). These findings corresponded to a Deauville score of 5, indicative of metabolically active, progressive disease (Fig. 4A and B). Following this, her case was discussed in a multi-disciplinary meeting, and a repeat biopsy of the adrenal mass was recommended. This was performed and was consistent with the original diagnosis of CD20-negative DLBCL, with a proliferative index of 90%. She was clinically quite symptomatic with a declining performance status. Based on this, a detailed discussion was carried out regarding the optimal treatment as she would not be able to tolerate an intensive chemotherapy regimen. Second-line chemotherapy with GDP (Gemcitabine, Dexamethasone, and Cisplatin) was planned, however the patient and their family refused further treatment.

## Discussion

PALs are exceptionally rare, and their presentation poses significant diagnostic challenges [2]. In this case, the patient’s presentation with bilateral adrenal masses was particularly challenging. Adrenal involvement in lymphoma is uncommon and adds complexity to the diagnosis. The lesions’ hypo-dense nature on CT and high SUV values on PET scans, combined with clinical symptoms and laboratory findings, guided the diagnosis of lymphoma.

The pattern of hypermetabolic adrenal lesions on PET-CT necessitates a differential diagnosis. Adrenal cortical carcinoma typically presents with heterogeneous FDG uptake, irregular margins, necrosis, and possible venous invasion. Metastases from primaries such as lung, breast, or melanoma may also show variable uptake, often bilateral but less commonly isolated to the adrenal glands. Pheochromocytomas demonstrate variable but often intense uptake, requiring correlation with biochemical markers. Infectious etiologies such as adrenal tuberculosis may show mild to moderate uptake, usually accompanied by calcification or adjacent lymphadenopathy. In contrast, primary adrenal lymphoma usually presents with bilateral, homogeneously FDG-avid lesions, without necrosis or calcification. In this case, the absence of necrosis, bilateral involvement, and high homogeneous FDG uptake supported the diagnosis of lymphoma over other possibilities. Prompt image-guided biopsy was performed based on these findings, leading to early histological confirmation of lymphoma. Imaging also played a central role in guiding treatment decisions, with interim PET-CT after 4 cycles of CHOP and intrathecal methotrexate informing the continuation of therapy due to partial metabolic response, and end-of-treatment PET-CT detecting disease progression, necessitating consideration of salvage chemotherapy.

CD20, a transmembrane protein encoded by MS4A1 gene on chromosome 11 and crucial for B-cell development and activation, is expressed on most B-cells [5,8]. Certain genetic mutations can alter CD20, leading to its absence in some aggressive lymphomas [9]. This loss is linked to poor outcomes and resistance to standard treatments, along with extra nodal involvement [8].

CD20-negative DLBCLs comprise a varied group of lymphomas characterized by a broad spectrum of clinical and histological features, with several subtypes [10]. Of these subtypes, plasmablastic lymphoma (PBL) comprises about 75% of the cases, whereas the other subtypes are rarer and commonly persist with an HIV and EBV coinfection [11,12]. Some exceptionally rare CD20-negative DLBCLs have been documented that do not align with the characteristics of any known subtype [11].

In our case, the histopathology showed large atypical lymphoid cells negative for CD20, consistent with a B-cell lineage as demonstrated by positive staining for CD79a and PAX5. This immune profile, combined with the absence of markers characteristic of the recognized CD20-negative DLBCL subtypes, underscored the unclassifiable nature of the CD20-negative DLBCL [13]. These unclassifiable CD20-negative DLBCLs pose significant diagnostic challenges due to their variable immuno-phenotypes and resistance to conventional therapies [12].

The therapeutic strategy for CD20-negative DLBCL is challenging due to the absence of standardized treatment protocols and the inability to use rituximab [10]. Our patient initially received the CHOP regimen combined with high-dose and intrathecal MTX to address potential central nervous system involvement, showing an initial partial response. However, subsequent PET scans indicated disease progression. This necessitated a repeat biopsy to recheck CD20 status, followed by consideration of second-line chemotherapy with GDP (Gemcitabine, Dexamethasone, and Cisplatin). Despite counseling on the possibility of salvage chemotherapy followed by autologous stem cell transplantation (auto-SCT), the patient and their family declined due to the poor prognosis and the patient's low ECOG performance.

CD20-negative DLBCLs often resist conventional therapies like CHOP, necessitating alternative regimens such as CODOX-M/IVAC, dose-adjusted EPOCH, and HyperCVAD [5]. Upregulating CD20 expression using agents like 5-azacytidine, which has shown effectiveness in CD20-negative B-cell acute lymphoblastic leukemia, presents another strategy [14]. Additionally, Bortezomib combined with dose-adjusted EPOCH has shown promise in treating PBL [7]. Epigenetic agents and the CXCR4 antagonist Plerixafor, which enhances rituximab-induced cell killing, might also offer potential benefits in managing CD20-negative lymphomas [15].

The diagnosis of CD-20 negative lymphomas requires pathologists to use a comprehensive panel of markers in order to identify B-cell lineage and reflect various stages of B-cell differentiation, including staining for CD79a, CD19, and PAX5 [16]. Notably, PAX5 serves as a reliable marker in CD20-negative DLBCLs, while other markers may be nonspecific [11,16].

## Conclusion

This case highlights the diagnostic and therapeutic challenges posed by PAL, particularly when it presents as CD20-negative DLBCL. The unclassifiable nature of this CD20-negative DLBCL further complicated management. While initial therapy with CHOP and high-dose methotrexate demonstrated a partial response, subsequent disease progression underscored the aggressive nature of this malignancy. The lack of effective treatment options for CD20-negative DLBCLs, coupled with the patient's declining clinical status, ultimately led to a poor outcome. This case emphasizes the need for continued research into the pathogenesis and optimal therapeutic strategies for such rare and clinically challenging lymphomas.

## Patient consent

We obtained written, informed consent for publication of this case from the patient.
